# Therapeutic hypercapnia reduces blood–brain barrier damage possibly via protein kinase Cε in rats with lateral fluid percussion injury

**DOI:** 10.1186/s12974-019-1427-2

**Published:** 2019-02-13

**Authors:** Wan-Chao Yang, Qi Wang, Lai-Ting Chi, Yue-Zhen Wang, Hong-Ling Cao, Wen-Zhi Li

**Affiliations:** 1 Department of Anesthesiology, Second Affiliated Hospital of Harbin Medical University, Harbin, China; 2 Anesthesiology Key Laboratory, Education Department, Harbin Medical University, No. 246 Xuefu Road, Harbin, 150086 China

**Keywords:** Hypercapnia, Traumatic brain injury, Protein kinases, Blood–brain barrier

## Abstract

**Background:**

This study investigated whether therapeutic hypercapnia (TH) ameliorated blood–brain barrier (BBB) damage and improved the neurologic outcome in a rat model of lateral fluid percussion injury (FPI), and explored the possible underlying mechanism.

**Methods:**

Rats underwent lateral FPI and received inhalation of 30%O_2_–70%N_2_ or 30%O_2_–N_2_ plus CO_2_ to maintain arterial blood CO_2_ tension (PaCO_2_) between 80 and 100 mmHg for 3 h. To further explore the possible mechanisms for the protective effects of TH, a PKC inhibitor staurosporine or PKCαβ inhibitor GÖ6976 was administered via intracerebral ventricular injection.

**Results:**

TH significantly improved neurological function 24 h, 48 h, 7 d, and 14 d after FPI. The wet/dry ratio, computed tomography values, Evans blue content, and histological lesion volume were significantly reduced by TH. Moreover, numbers of survived neurons and the expression of tight junction proteins (ZO-1, occludin, and claudin-5) were significantly elevated after TH treatment at 48-h post-FPI. TH significantly increased the expression of protein kinase Cε (PKCε) at 48-h post-FPI, but did not significantly change the expression of PKCα and PKCβII. PKC inhibitor staurosporine (but not the selective PKCαβ inhibitor-GÖ6976) inhibited the protective effect of TH.

**Conclusions:**

Therapeutic hypercapnia is a promising candidate that should be further evaluated for clinical treatment. It not only protects the traumatic penumbra from secondary injury and improves histological structure but also maintains the integrity of BBB and reduces neurologic deficits after trauma in a rat model of FPI.

## Background

Traumatic brain injury (TBI), a leading cause of disability and death worldwide, poses a substantial burden on health outcomes and expenditure. In the USA, 1.7 million people per year suffer from TBI and 50,000 of these people die [1, 2]. TBI basically consists of two phases: the initial injury that occurs at the time of the traumatic impact and the secondary injury that activates cascades of endogenous auto-destructive biochemical processes following initial injury. Secondary injury lasts over hours to days and involves many cellular processes such as blood–brain barrier (BBB) breakdown, apoptosis, oxidative stress, neuronal excitability, inflammation, and immune responses [3–5]. Secondary injury is regarded as a major cause for long-term post-TBI neurological dysfunction and death [6]. Furthermore, increasing size of injury around the contusion called “traumatic penumbra” is caused by the secondary injury [7]. Since secondary injury represents a region of tissue that is most affected by clinical management and neuroprotective interventions, it suggests a potential therapeutic window and a therapy target. Standard procedures for TBI therapy include hyperventilation, hyper-osmotic fluid treatment, and decompressive craniectomy. Unfortunately, to date, no effective therapies can be applied immediately after TBI to ameliorate the secondary damage and improve clinical outcomes.

Accumulating data show that the permeability of BBB serves as a key factor to mediate secondary brain damage, thus improving BBB integrity optimizes TBI treatment [6, 8, 9]. Protein kinase C (PKC) has been demonstrated to play a pivotal role in modulating microvascular permeability [10] and thus serves as a critical factor in BBB structural and functional integrity in pathological conditions [11–13]. Three important PKC isozymes—PKCα, PKCβ, and PKCε—directly modify components of the tight junction (TJ) complex to regulate microvascular permeability [14]. PKCα or PKCβ signaling may enhance TJ opening while PCKε has been shown to be associated with BBB maintenance [15–17].

Hypercapnia exerts neuroprotective effects following cerebral ischemic reperfusion, possibly via suppression of AQP4 and anti-apoptosis mechanisms [18–20]. Zhou et al. [21] reported that mild to moderate hypercapnia (PaCO_2_, 60–100 mmHg) does not significantly increase intracranial pressure (ICP) for the treatment of global cerebral ischemia in adult rats. Therefore, we hypothesize that hypercapnia may have a potential protective effect on BBB function against TBI.

In this study, we aimed to evaluate the therapeutic efficacy of hypercapnia on BBB function in rats with fluid percussion injury (FPI) and to explore whether hypercapnia regulated specific PKC isozymes against BBB disruption following TBI.

## Methods

### Animals and ethics approval

Adult male Sprague–Dawley rats (weighing 280–320 g) were obtained from the Second Affiliated Hospital of Harbin Medical University (Harbin, P. R. China). Animals were housed in standard cages, given free access to food and water, and kept in a 12-h day/night cycle. The experimental protocols were approved by the Institutional Animal Care Committee of Harbin Medical University, and all procedures were conducted in strict accordance with the guidelines for the care and use of laboratory animals of Harbin Medical University as well as the ARRIVE (Animal Research: Reporting In Vivo Experiments) guidelines for animal research.

### Experiment procedure

Each rat was anesthetized via intraperitoneal injection of 30 mg/kg pentobarbital. All rats were intubated and received mechanical ventilation with a gas mixture of 30% O_2_–70% N_2_ using a Harvard small animal ventilator 683 (Natick, MA, USA). The tidal volume was 9 ml/kg, and the respiratory rate was 45 breaths/min with a 1:1 inspiratory to respiratory ratio. The left femoral artery was cannulated with a 24-gauge (G) catheter for recording the mean arterial pressure (MAP), heart rate (HR), and blood gas analysis (PaO_2_, PaCO_2_, pH). The left femoral vein was cannulated for injection of pentobarbital (3 mg/kg per hour) and Evans blue dye. ICP was continuously measured via a 4-mm-long catheter (22 G) placed into the lateral ventricle (1 mm posterior to the bregma, 1.5 mm right of the sagittal suture).

### Experimental groups

Rats were randomly assigned (by drawing lots) to five groups (*n* = 24 per group):

1. Sham group rats received drilled craniotomy and were placed on the FPI device without injury. During the following 3 h period, rats were mechanically ventilated with a gas mixture of 30% O_2_–70% N_2_.
2. Trauma group (group T): the FPI model was established followed by mechanical ventilation with a gas mixture of 30% O_2_–70% N_2_ for 3 h.
3. Hypercapnia group (group T+H): the FPI model was established followed by mechanical ventilation with a gas mixture of 30%O_2_–N_2_–CO_2_ for 3 h. The gas flow of N_2_ and CO_2_ was continuously adjusted to maintain PaCO_2_ between 80 and 100 mmHg based on the arterial blood gas analysis.
4. PKC inhibitor staurosporine group (group T+H+PKCi): rats received the same treatment procedure as rats in group T+H, except that they were given intraventricular injection of 5 μL staurosporine (0.1 μM) (Santa Cruz, CA, USA) immediately after the FPI was established.
5. PKCαβ inhibitor group (group T+H+PKCαβi): rats were treated with the same procedure as rats in group T+H, except that they were given intraventricular injection of 5 μL PKCαβ inhibitor GÖ6976 (1 μM) (ab141413; Abcam, Cambridge, UK) immediately after the FPI was established.

In each group, six rats were used for the Evans blue test, six for Nissl staining, six for wet/dry ratio calculation, six for Western blot, six for PCR test, six for CT scanning, and six for behavior test scoring.

### Establishment of lateral fluid percussion injury model

FPI was established using the fluid percussion device (FP 302, AmScien Instruments, Richmond, VA, USA) as previously described [22–24]. Rats were fixed into a stereotaxic frame (Narishige, Scientific Instrument Lab, Tokyo, Japan). A 4-mm-diameter craniotomy was made 2 mm posterior to the bregma and 2.5 mm left to the sagittal suture, using a dental drill. The dura was exposed intact, and care was taken to avoid any damage to the blood vessels in the dura. The dura was connected to a stainless-steel female Leur-Lot fitting. Dental acrylate was used to fill the space between the dura and the fitting. The pendulum bob was placed at 17° and was later released to produce an impact pressure of between 1.5 and 2 atm. This pressure range induced a moderate brain injury [23]. Rats were then removed from the device and placed on a warming pad to maintain normal body temperature. The scalp was sutured, and animals were ventilated for 3 h. The intubation tube was removed after the rats recovered spontaneous breathing.

### Administration of PKC inhibitor

After the FPI model was established, rats were fixed into a stereotaxic frame. Injection of 5 μL staurosporine (0.1 μM) or GÖ6976 (1 μM) into the lateral ventricle (0.8 mm posterior to the bregma and 1.5 mm lateral to the sagittal suture, 4 mm deep) was performed.

### Measurement of brain edema

Forty-eight hours after FPI, each rat was anesthetized and then decapitated; the brain was removed. The injured hemisphere was weighed and recorded as wet weight (*W*). Next, the brain was dried at 80 °C for 48 h and then remeasured as dry weight. The *W*/*D* ratio was calculated by an investigator who was blinded to the grouping according to the following equation:

*W*/*D* ratio = [(wet weight – dry weight)/wet weight] × 100% [25].

### Computed tomography scanning

Forty-eight hours after FPI, the rats were anesthetized. For brain computed tomography, CT images were acquired using a whole-body CT scanner (GE LightSpeed VCT, GE Healthcare, Chicago, IL, USA). The rats’ brains were scanned using 140 kVp, 400 mA, and an exposure time of 2 s. Data were reconstructed in real time using a Dose system (GE Healthcare). Edema was defined as CT values less than 20 HU, while the CT values in the normal brain tissue varied between 25 and 40 HU. Scanning was started from the border of cerebral lesion, and CT images were acquired with a thickness of 0.625 mm. CT values of six sequential slices (with a lesion diameter of 3.75 mm) were required and compared by an investigator who was blinded to the protocol.

### Evans blue detection

Evans blue (EB) dye was used to assess the permeability of BBB and was performed and analyzed by a grouping-blinded researcher. EB dye (2%, 5 ml/kg) was injected through the left femoral vein 3 h after FPI establishment. One hour after EB injection, rats were transcardially perfused with normal saline for 10 min. The brain of each rat was then removed, and the left prefrontal cortex was separated, weighed, and homogenized in 50% trichloroacetic acid (TCA). Homogenates were centrifuged for 10 min, and the supernatant was collected. EB dye was measured using an absorbance spectrophotometer at 620 nm. EB dye content was calculated and expressed as nanogram/milligram tissue as previously described [26].

### Nissl staining and measurement of lesion volume

Forty-eight hours after FPI, rats were euthanized by overdose anesthesia. Rats were then transcardially perfused with normal saline followed by 4% paraformaldehyde. Brains were removed, fixed in 4% paraformaldehyde for 24 h, and dehydrated in 30% sucrose in PBS at room temperature. Cerebral lesion was sequentially cut at 25 um thick, and 167 ± 16 slices were obtained for each rat. The slices were mounted and then stained with Nissl stain as previously described [27]. Nissl-stained sections were then photographed. At 40× magnification, five fields in each section of the peri-lesion were randomly chosen to count the number of neurons.

The Nissl-stained images of the whole brain section were obtained using a scanner (LaserJet Pro MFP m128fn). The lesion volumes were measured by a researcher blinded to the experimental conditions using Image-Pro Plus version 6.0 software (Media Cybernetics, Bethesda, MD, USA). The lesion area was obtained by bordering damaged or abnormal tissues in the ipsilateral cortex. The lower boundary of the lesion was outlined by the area in which the neuron density was less than normal. Edema area was calculated by ipsilateral hemisphere area minus contralateral hemisphere area. To eliminate the effects of edema, the percentage of lesion volume was calculated according to the following equation: [measured lesion area – edema area] / [(ipsilateral hemisphere area + contralateral hemisphere area) – edema area]. The lesion of each slice was calculated, and the average of the whole brain was recorded and compared by a researcher who was blinded to the experiment protocol.

### Western blot analysis

The peri-lesion part of the ipsilateral hemisphere, defined as “penumbra,” was removed and immediately stored at − 80 °C and later used for Western blot analysis as previously described [28] at 48 h post-FPI. Protein samples from the left prefrontal cortex were dissolved in lysis buffer and protease inhibitor cocktail (Sigma) at 4 °C for 1 h. After centrifugation for 10 min at 12,000×*g*/min, the supernatant was collected and the protein concentration was calculated by BCA protein measurement kit (Bio-Rad). Equal amount of proteins were loaded in SDS-PAGE gel, and the products of the electrophoresis were transferred to PVDF membrane (Invitrogen) following the wet transfer procedure (transfer buffer: 25 mM Tris, 192 mM glycine, 20% methanol). The membrane was blocked with 5% nonfat milk in TBST buffer and then incubated with primary antibodies overnight at 4 °C. The primary antibodies (1:1000 dilution) against PKCα (sc-8393; Santa Cruz Biotechnology, Dallas, TX, USA); PKCβII (sc-13,149; Santa Cruz Biotechnology); PKCε (sc-1681, Santa Cruz Biotechnology); ZO-1 (sc-33,725; Santa Cruz Biotechnology); occludin (sc-133,256; Santa Cruz Biotechnology); and claudin-5 (sc-28,670; Santa Cruz Biotechnology) were used. After extensive rinsing in TBST buffer, the membranes were incubated with HRP-conjugated secondary antibody (diluted at 1:5000, Rockland Inc., Gilbertsville, PA, USA) at 37 °C for 1 h. The final product was viewed using chemiluminescent substrates by a grouping-blinded researcher. The expression level of β-actin was used as loading control.

### Reverse transcription-quantitative polymerase chain reaction

Total RNA was extracted from peri-lesion tissues using TRIzol reagents (Invitrogen, Carlsbad, CA, USA). According to the previously published description of the reverse transcription-quantitative polymerase chain reaction (RT-qPCR) [29], 1 μg of total RNA was used for reverse transcription with PrimeScript RT Enzyme Mix I (Takara Bio Inc., Otsu, Shiga, Japan) and a reverse transcription primer.

cDNA solution (1 μL) was mixed with SYBR PrimeScript Ex Taq II (Takara Bio Inc.) and the primers in a total volume of 20 μl. The result was analyzed with a LightCycler 2.0 (Roche, Basel, Switzerland) and an investigator who was blinded to grouping applied the 2^△△−Ct^ method to calculate the relative quantity of PKCε mRNA. Glyceraldehyde-3-phosphate dehydrogenase (GAPDH) mRNA was used as internal control (forward primer, 5′-CTCTGCTCCTCCTGTTCGAC-3′, and reverse primer, 5′-TTAAAAGCAGCCCTGGTGAC-3′). The primers for qPCR were designed on the basis of previous data: forward primer 5′-TACGAAGTGCGCTGGGCTAA-3′ and reverse primer 5′-GGAGCCACAGTGGTCACAGAA-3′.

### Behavioral testing

The Modified Neurological Severity Score (mNSS) was used to evaluate neurologic function as previously described by Chen et al. [30]. mNSS tests were performed before FPI; and at 24 h, 48 h, 7 d, 14 d, and 28 d after FPI, by an investigator blinded to the experimental condition. This 18-score system consisted of motor, sensory, reflex, and beam balance tests. A higher score indicates a more severe neurologic impairment.

### Statistical analysis

Statistical analyses were performed using SAS version 9.3 software (SAS Institute Inc., Cary, NC, USA). Data are expressed as mean ± standard error of Measurement (SEM). The results from physiological variables and biochemical and animal behavior studies were examined by repeated measures analysis of variance (ANOVA). We selected the mixed effect model to analyze it: after the interaction effect was statistically significant, single-effect comparison was performed by the “contrast program” in SAS software. Using statistician programming and setting of the error, we corrected the *p* value using code. A *p* value < 0.05 was considered significant for all statistical analyses in this study.

## Results

### Physiologic parameters

We first examined the physiological parameters in the sham group, group T, and group T+H. Table 1 showed the changes in ICP, MAP, PaO_2_, PaCO_2_, and pH after FPI with hypercapnia. ICP in the sham group did not change significantly during the experiment. FPI resulted in a significant increase in ICP, peaked at 68.00 ± 0.76 mmHg in group T and 67.00 ± 0.65 mmHg in group T+H rats and gradually dropped to a normal level within 30 min. ICP did not increase significantly during 3 h of CO_2_ inhalation and was kept within physiological values in the three groups.

**Table 1:** Physiologic parameters

	Group	Time								
		Baseline	Trauma	CO_2_ inhalation						
				15 min	30 min	60 min	90 min	120 min	150 min	180 min
ICP (mmHg)	Sham	14.50 ± 0.46	14.25 ± 0.34	15.75 ± 0.60	15.00 ± 0.29	15.25 ± 0.45	14.50 ± 0.46	14.75 ± 0.34	14.50 ± 0.46	14.50 ± 0.20
	T	14.75 ± 0.53	68.00 ± 0.76^#^	17.25 ± 0.53	17.00 ± 1.26	17.25 ± 0.93	18.25 ± 1.36^#^	19.50 ± 1.93^#^	18.75 ± 1.17^#^	19.25 ± 0.97^#^
	T+H	13.50 ± 0.61	67.00 ± 0.65^#^	21.25 ± 0.60*^#^	18.50 ± 1.06^#^	16.00 ± 1.61	14.50 ± 0.94*	14.50 ± 1.10*	15.50 ± 1.02*	15.00 ± 0.76*
MAP (mmHg)	Sham	106.89 ± 2.81	106.89 ± 2.76	104.09 ± 3.32	104.78 ± 3.61	106.33 ± 2.29	106.50 ± 1.76	110.61 ± 2.30	105.72 ± 3.29	106.48 ± 4.48
	T	99.56 ± 3.57	163.76 ± 5.15^#^	90.61 ± 3.18^#^	91.22 ± 4.0^#^	94.83 ± 2.05^#^	97.00 ± 1.96	102.39 ± 4.72	99.89 ± 5.92	97.06 ± 5.06
	T+H	102.89 ± 2.51	163.38 ± 5.22^#^	87.22 ± 3.74^#^	89.33 ± 2.12^#^	97.83 ± 4.08	100.28 ± 3.37	100.42 ± 3.49	101.94 ± 2.66	100.74 ± 2.64
PaO_2_ (mmHg)	Sham	103.42 ± 2.6		110.75 ± 2.42	113.22 ± 2.20	114.5 ± 2.42	112.62 ± 2.78	112.82 ± 1.64	113.85 ± 2.12	113.67 ± 2.11
	T	108.87 ± 3.69		104.58 ± 5.00	111.78 ± 4.16	110.72 ± 3.55	109.10 ± 5.0	111.37 ± 2.93	109.38 ± 2.44	108.55 ± 3.68
	T+H	104.83 ± 3.32		118.82 ± 5.23*	130.28 ± 3.08*^#^	138.78 ± 4.10*^#^	138.30 ± 6.28*^#^	141.40 ± 7.00*^#^	138.65 ± 3.36*^#^	146.87 ± 3.65*^#^
PaCO_2_ (mmHg)	Sham	37.10 ± 1.18		39.97 ± 1.67	38.38 ± 1.52	37.20 ± 0.73	39.07 ± 1.07	39.58 ± 1.47	42.45 ± 1.51	39.93 ± 1.31
	T	37.33 ± 2.01		41.90 ± 2.85	39.83 ± 2.06	39.93 ± 1.72	40.67 ± 2.97	37.92 ± 2.44	38.48 ± 2.73	39.20 ± 2.47
	T+H	34.05 ± 1.83		72.53 ± 8.15*^#^	84.25 ± 4.21*^#^	89.97 ± 4.07*^#^	97.78 ± 2.29*^#^	94.60 ± 4.43*^#^	92.48 ± 3.98*^#^	91.58 ± 3.56*^#^
pH	Sham	7.38 ± 0.02		7.38 ± 0.02	7.40 ± 0.01	7.39 ± 0.02	7.38 ± 0.01	7.43 ± 0.01	7.40 ± 0.02	7.38 ± 0.01
	T	7.41 ± 0.02		7.36 ± 0.01	7.37 ± 0.02	7.39 ± 0.02	7.35 ± 0.02	7.34 ± 0.02	7.33 ± 0.02	7.37 ± 0.02
	T+H	7.39 ± 0.01		7.15 ± 0.01*^#^	7.06 ± 0.04*^#^	7.07 ± 0.02*^#^	7.05 ± 0.02*^#^	7.06 ± 0.01*^#^	7.08 ± 0.02*^#^	7.09 ± 0.02*^#^

ICP, MAP, PaO_2_, PaCO_2_ and pH in the sham group, group T, and group T+H. Data were expressed as means ± SEM. *n* = 6

^#^*p* < 0.05 vs. sham, *p < 0.05 vs. group T

MAP significantly elevated immediately after trauma in both group T and group T+H, compared with the control group (*p* < 0.05), MAP elevation lasted for 60 min in group T and 30 min in group T+H. The fluctuation of MAP was believed to be in response to ICP changes.

PaCO_2_ in group T+H rats was maintained within the range of 80–100 mmHg during 3-h CO_2_ inhalation, and was significantly higher compared with the sham group (*p* < 0.05). For PaO_2_, compared with group T, hypercapnia significantly increased PaO_2_ as early as 15 min post-PFI, which lasted for 3 h (*p* < 0.05). pH values in group T+H were significantly lower compared with the other two groups (*p* < 0.05).

### Hypercapnia ameliorates cerebral edema after FPI

**Fig. 1:**
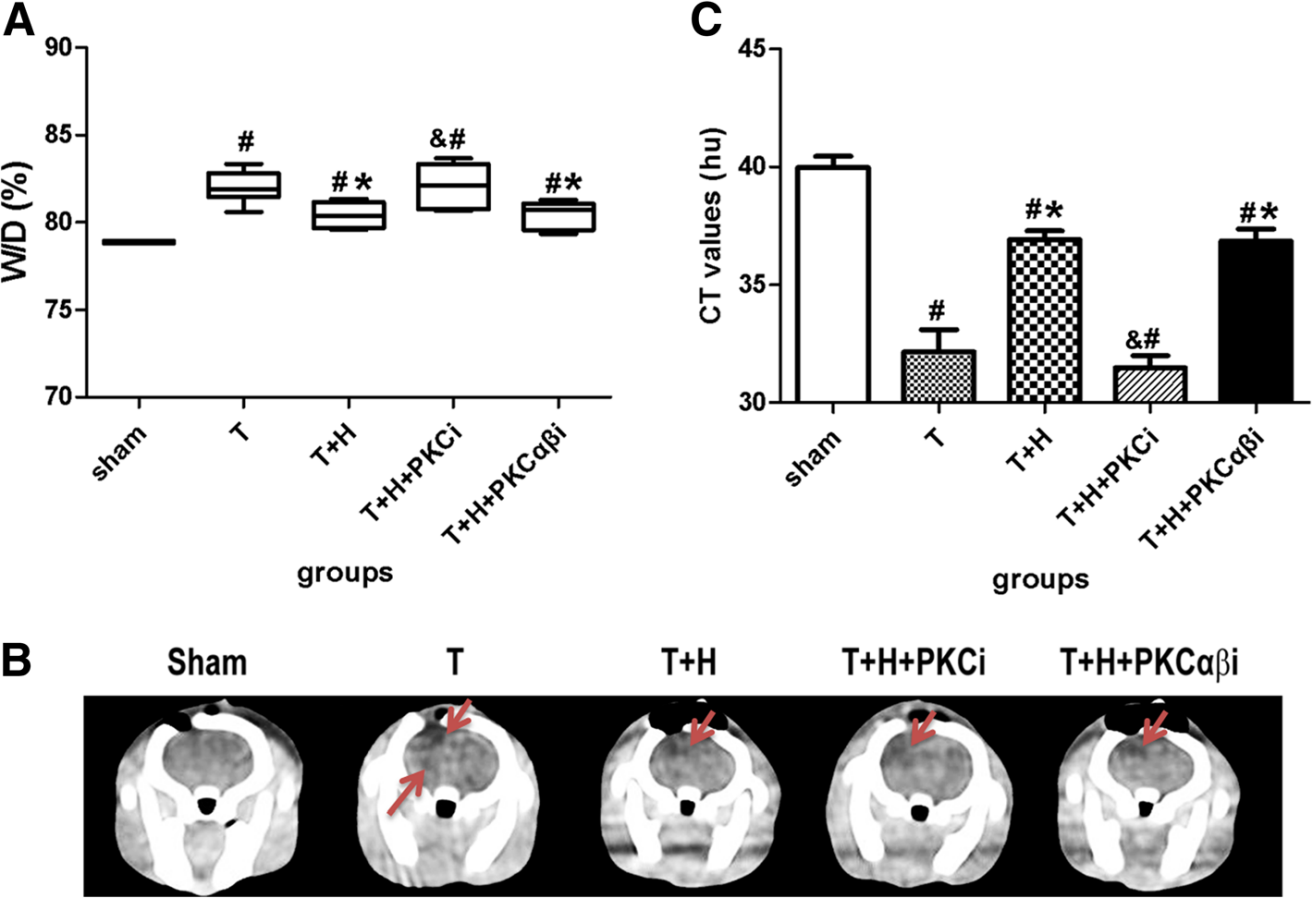
Hypercapnia reduces brain edema. **a** The wet/dry (*W*/*D*) ratio of the injured brain of rats in the sham group, group T, group T+H, group T+H+PKCi, and group T+H+PKCαβi rats at 48-h post-FPI. **b** Representative computed tomography images of coronal section of rats in the sham group, group T, group T+H, group T+H+PKCi, and group T+H+PKCαβi rats at 48-h post-FPI. The darker areas with lower CT values represented cerebral edema. **c** CT values. The red arrow indicates post-traumatic brain cerebral edema**.** Data were expressed as means ± SEM. *n* = 6. ^#^*p* < 0.05 vs. sham, *p < 0.05 vs. group T, ^&^*p* < 0.05 vs. group T+H

At 48-h post-FPI, the W/D ratio in group T rats was significantly increased compared with the sham group (*p* < 0.05, Fig. 1a). The *W*/*D* ratio was significantly reduced in group T+H compared with the group T rats (*p* < 0.05, Fig. 1a), suggesting that hypercapnia ameliorated cerebral edema. PKCi (but not PKCαβi) significantly inhibited hypercapnia-induced decrease in cerebral edema (*p* > 0.05) (Fig. 1a).

Similarly, CT values was significantly decreased in group T compared with the sham group, suggesting that PFI induced cerebral edema, The CT values in group T+H rats were significantly higher than those in group T rats. PKCi (but not PKCαβi) significantly inhibited hypercapnia-induced increase in CT values (*p* < 0.05) (Fig. 1b, c).

### The permeability of the blood–brain barrier was ameliorated by hypercapnia

**Fig. 2:**
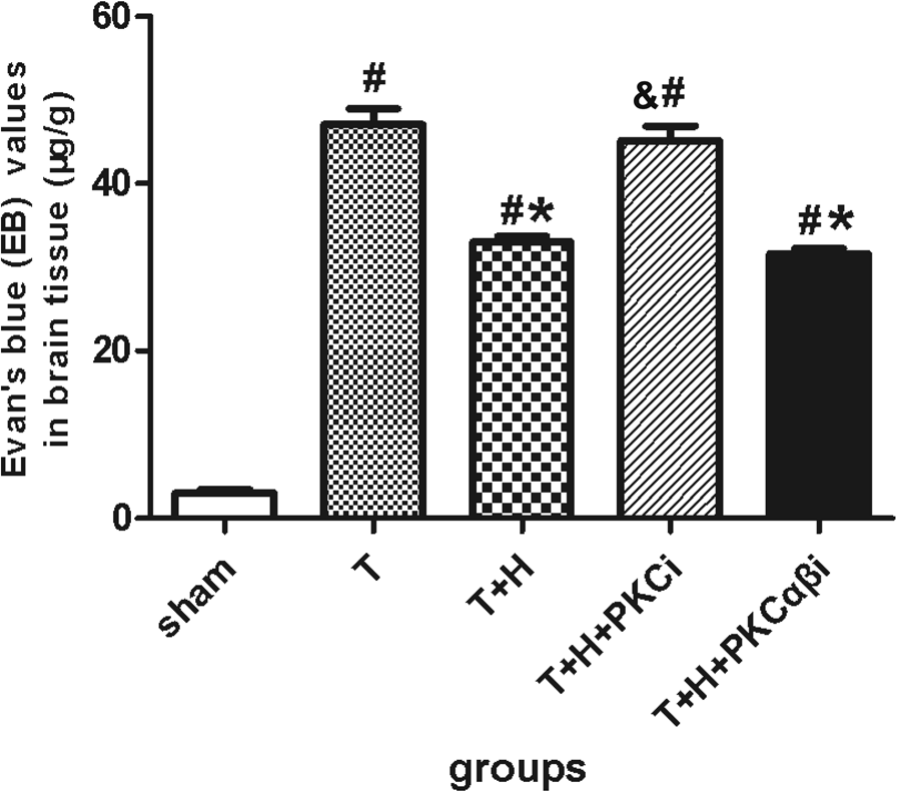
Evans blue values of the injured brain from rats in the sham group, group T, group T+H, group T+H+PKCi, and group T+H+PKCαβi rats at 3-h post-FPI. Data were expressed as means ± SEM. *n* = 4. ^#^*p* < 0.05 vs. sham, *p < 0.05 vs. group T, ^&^*p* < 0.05 vs. group T+H.

EB extravasation in the injured prefrontal cortex was significantly increased 3 h after FPI in group T rats. Hypercapnia significantly ameliorated EB extravasation (*p* < 0.05). PKCi (but not PKCαβi) significantly blocked the effect of hypercapnia (Fig. 2).

### Hypercapnia reduced lesion volume and improved neuronal survival

**Fig. 3:**
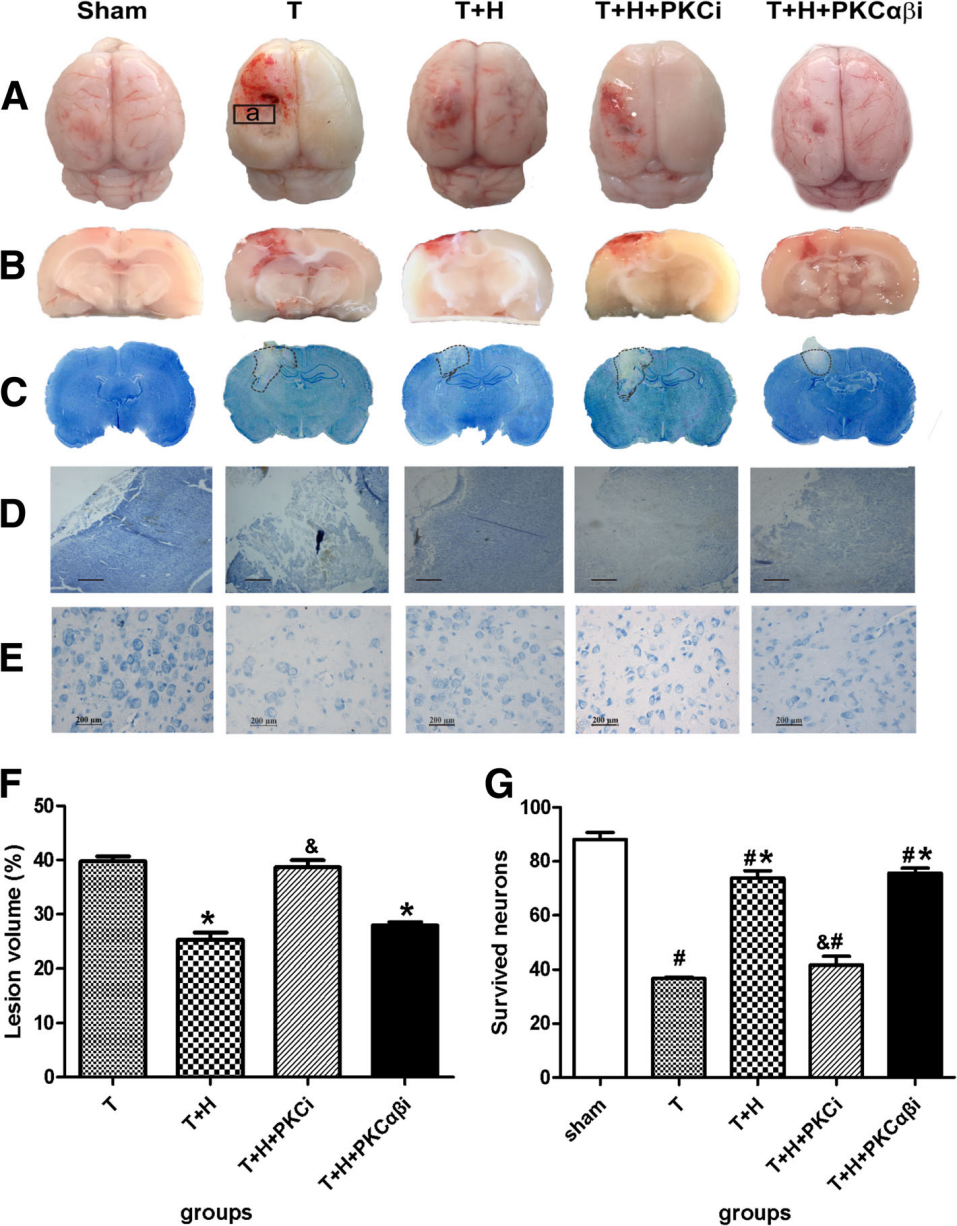
Hypercapnia reduced lesion volume and improved neuronal survival. **A**, **B** The whole brain (**A**) and coronal section of the injured brain (**B**) in the sham group, group T, group T+H, group T+H+PKCi, and group T+H+PKCαβi rats at 48-h post-FPI. a, the peri-lesion part (“penumbra”). **C**, **D** Representative images showing outlined lesion area (**C**) and Nissl staining (×4 (**D**) and ×40 (**E**)). Scale bars: *D* = 2000 μm, *E* = 200 μm. **F** Lesion volume was calculated and analyzed. **G** Survived neurons in the penumbra. *n* = 4. ^#^*p* < 0.05 vs. sham, *p < 0.05 vs. group T, ^&^*p* < 0.05 vs. group T+H

Hypercapnia resulted in a significant decrease in lesion volume and a significant increase in survived neurons at 48 h post-FPI (*p* < 0.05, Fig. 3). A significant increase in lesion volume and a significant decrease in survived neurons was observed in group T+H+PKCi compared with those in group T+H (*p* < 0.05) rats. However, PKCαβ treatment did not result in significant changes in lesion volume and neuronal survival compared with group T+H rats (*p* < 0.05) (Fig. 3).

### Hypercapnia resulted in an increase in the expression of tight junction proteins and a significant increase in the expression of PKCε

**Fig. 4:**
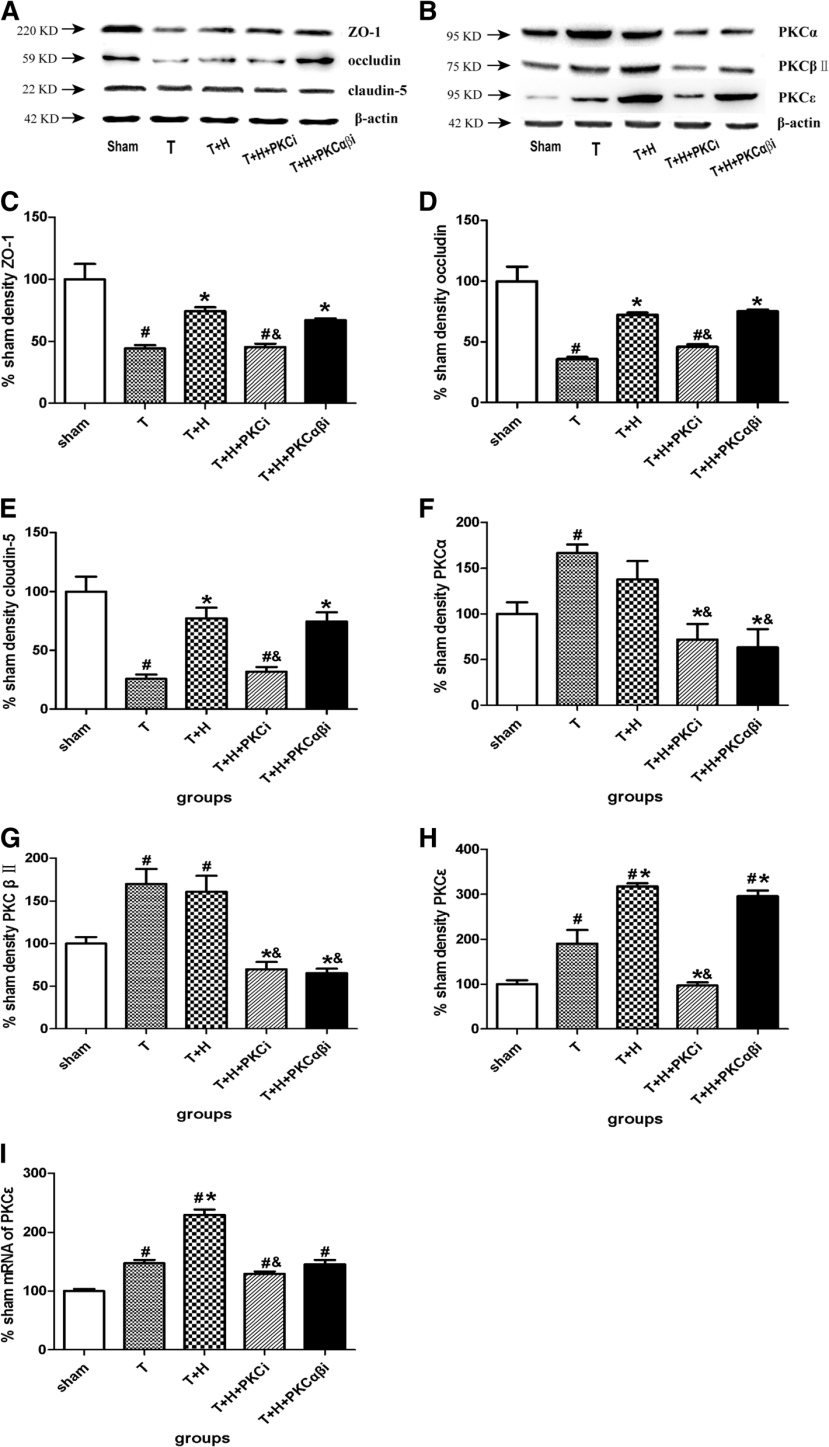
Hypercapnia increased the expression of TJ proteins and PKCε*.* **a**, **b** Representative Western blot results showing the expression of the tight junction proteins ZO-1, occludin, and claudin-5 (**a**) and PKCα, PKCβII, and PKCε (**b**) in the sham group, group T, group T+H, group T+H+PKCi, and group T+H+PKCαβ rats at 48-h post-FPI. **c**–**h** Quantification of the expression of ZO-1 (**c**), occludin (**d**), claudin-5 (**e**), PKCα (**f**), PKCβII (**g**), and PKCε (**h**) in the sham group, group T, group T+H, group T+H+PKCi, and group T+H+PKCαβi rats. **i** RT-PCR results showing the mRNA expression of PKCε in the sham group, group T, group T+H, group T+H+PKCi, and group T+H+PKCαβI rats. Data were expressed as means ± SEM. *n* = 4. ^#^*p* < 0.05 vs. sham, *p < 0.05 vs. group T, ^&^*p* < 0.05 vs. group T+H

The expression of TJ proteins and PKC isozymes were shown in the Fig. 4a and b. Hypercapnia increases the expression of ZO-1 (Fig. 4c), occludin (Fig. 4d), and claudin-5 (Fig. 4e). PKCi (but not PKCαβi) blocked hypercapnia-induced upregulation of ZO-1, occludin, and claudin-5. After FPI, the expression levels of PKCα (Fig. 4f), PKCβII (Fig. 4g), and PKCε (Fig. 4h) were significantly increased, while hypercapnia only significantly elevated the expression of PKCε (Fig. 4h). PKCi decreased the expression of all the three PKCs and PKCαβi inhibited the expression of PKCαβ.

mRNA of PKCε was detected by RT-PCR. Hypercapnia significantly increased the mRNA expression of PKCε. However, only PKCi (but not PKCαβi) inhibited the mRNA expression of PKCε (*p* < 0.05) (Fig. 4i).

### Hypercapnia improved mNSS scores

**Fig. 5:**
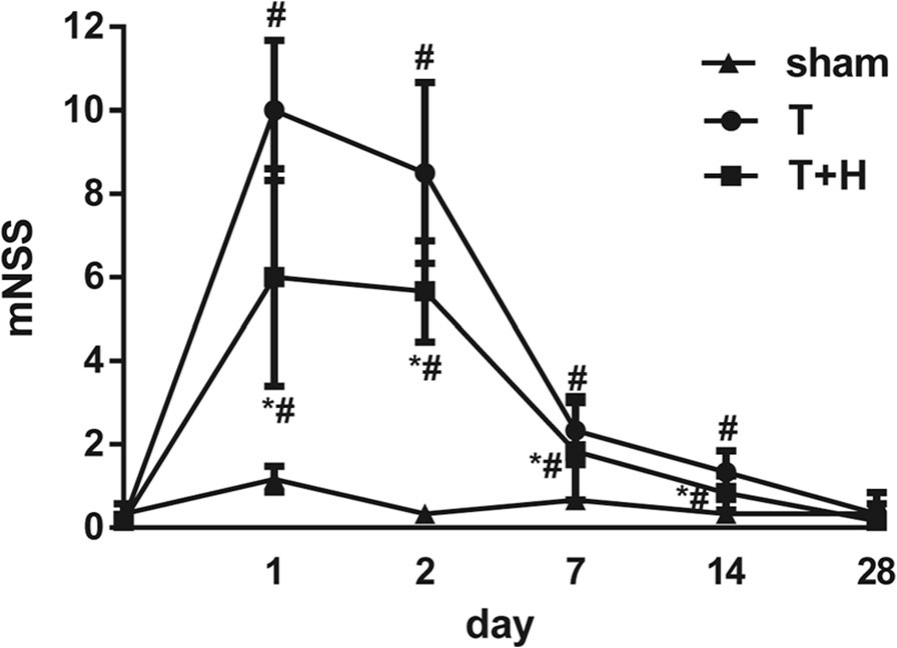
The Modified Neurological Severity Score (mNSS) in the sham group, group T, and group T+H rats at 1, 2, 7, 14, and 28 days post-FPI. Data were expressed as means ± SEM. *n* = 6. ^#^*p* < 0.05vs.sham, *p < 0.05 vs. group T

At day 1, day 2, day 7, and day 14 after FPI, mNSS scores in group T rats were elevated compared with those in the sham group rats (*p* < 0.05). The neurological dysfunction of group T rats recovered within 28 days after FPI. mNSS scores in group T+H rats were significantly reduced at days 1, 2, 7, and 14 after FPI compared with group T rats (*p* < 0.05), and there were no significant differences between the two groups at day 28 (*p* > 0.05) (Fig. 5).

## Discussion

Hypercapnia was first found to produce neuroprotection against hypoxic ischemic injury in immature animal models [31, 32]. Later, hypercapnia has been reported to reduce neurologic deficit and ameliorate histopathologic damage and cerebral apoptosis in global cerebral ischemia [33]. In addition, hypercapnia improves functional recovery in focal cerebral ischemia and increases the 28-day survival rate [18, 34].

Penumbra is defined as a region of intermediate cerebral blood flow depression surrounding the densely ischemic core in stroke [35]. Traumatic penumbra may need to be defined physiologically rather than confined in terms of perilesional anatomy, is most vulnerable to secondary ischemic injury, and can be treated by therapeutic interventions [7, 35, 36]. TBI has similar pathophysiology to ischemic injury, including BBB interruption, edema, neuronal loss, apoptosis, and cognitive dysfunction. This study served as the first study to use therapeutic hypercapnia (TH) for the treatment of TBI in rats and showed for the first time how hypercapnia exerted neuroprotective effects on TBI in rats—by reducing brain edema, improving BBB function, decreasing lesion volume, and improving neurological outcome.

PKC family is widely expressed in tissues and consists of at least 12 isozymes, including conventional (a, βΙ, βII, and γ); novel (δ, ε, η, θ, μ, ѵ); and atypical (ζ, ɩ) isoforms with distinct distribution and different sensitivity to Ca^2+^, and regulation of diverse signal transduction and cellular activities [37, 38].

Among these isozymes, three important PKCs—PKCα, PKCβII, and PKCε—were shown to be most revolved in the regulation of the expression of TJ proteins. There are several possible mechanisms by which PKC may be altering BBB permeability. One possible mechanism is direct regulation (phosphorylation) of the TJ proteins by PKC. PKCα and PKCβ have both been implicated in regulating cell permeability after ischemia and inflammatory stimuli [10]. PKCε was found to play a beneficial role in the regulation of TJ proteins, while PKCβII aggravated the disruption of TJ proteins during aglycemic hypoxia [15]. In addition, increased PKCα activity exacerbates neuronal damage after poststroke brain injury [39].

We found that FPI increased the expression of injurious PKCα and PKCβII in groups T and T+H compared with sham, as well as the beneficial PKCε. Although our results do not show an increase in PKCα and β-expression by hypercapnia treatment compared to group T, our results do not exclude the possibility that PKCα and β and other isozymes may play a role in altering BBB endothelial cell integrity during FPI. In addition, our data, which show an increase in PKCε expression in group T+H, suggest that this may be this one isozyme that is important in regulating endothelial barrier integrity by hypercapnia. In addition, hypercapnia significantly increased the mRNA expression of PKCε at 48-hpost-FPI. This was accompanied by augmented expression of TJ proteins ZO-1, occludin, and claudin-5 as well as decreased brain edema. As the BBB is formed by endothelial cell TJs, the increased TJ proteins after hypercapnia suggests that BBB function were improved [14].

Furthermore, the non-selective PKC inhibitor staurosporine significantly decreases the expression of PKCα, PKCβII, and PKCε, and this blocked the protective effects of hypercapnia. GÖ6976 (an inhibitor of PKCα, PKCβ, and PKCγ but not PKCε) had no effect on hypercapnia-induced neuroprotection. These results suggest that hypercapnia may exert neuroprotective effects via upregulation of PKCε expression.

As noted, animal model of cerebral ischemic and TBI share certain comparable pathobiology. It has been reported that PKCε increased BBB integrity by upregulation of the expression of TJ proteins [11, 16, 40, 41]. In addition, PKCε activation protects BBB acutely and improves neuronal function chronically [28]. In cerebral ischemia models, PKCε activation improves neuronal protection and increases cerebral blood flow (CBF) after ischemia [42, 43]. In addition, PKCε activation plays a protective role in ischemic pre-conditioning (IPC) conditions [34, 44]. PKCε exerts its neuroprotective effect by activating adenosine-induced ATP-sensitive mitochondrial potassium (mKATP) channels, decreasing the production of ROS and calcium overload [45], preserving mitochondrial membrane potential, maintaining energy, and reducing calcium influx [46]. PKCε has also been found to be closely associated with IPC and *N*-methyl-d-aspartate (NMDA)-induced ischemic tolerance [47, 48]. We believe that this evidence suggests that PKCε serves as a key factor in protective effects against TBI.

Although we did not measure CBF in this study, our previous study found that hypercapnia treatment resulted in CBF recovery in rats treated with mild to moderate hypoxia (PaO_2_ > 50 mmHg) [20]. In addition, though in combination with acidosis and hyperoxia, carbon dioxide inhalation has been reported to cause vasodilation and increase CBF [49]. We speculate that in this rat model of FPI, hypercapnic acidosis appears to increase PaO_2_ through two mechanisms: increased PaCO_2_ and decreased pH leads to right shift of the O_2_-hemoglobin deviation curve and augmented oxygen delivery through increased cerebral blood flow (CBF). Hypercapnic acidosis also decreases the shunt of the lung and increases arterial oxygenation, which increases the cardiac output and decreases the oxygen consumption. These systemic effects may all explain better cerebral oxygenation and improved neurological outcome. Since PKCε modulates CBF in animals after ischemia [42], we could not exclude the possibility that hypercapnia produces cerebral protection via increasing CBF by upregulation of PKCε. In addition, PKCε upregulation by hypercapnia reduced neuronal loss and decreased apoptosis cells in this rat model of FPI (data not shown). This is consistent with a previous report showing that PKCε activation is responsible for anti-apoptosis by regulation of BCL-2 [50–52].

Together, these studies suggest that PKCε may be the key factor for hypercapnia-induced neuroprotection via different mechanisms including preserving the BBB, increasing CBF regulation, and inhibiting apoptosis. However, it remains unclear which PKC downstream signaling pathways (such as PKCε-CREB-Nrf2, PKCε/MAPK, PKCε/MERK, or PKC-mKATP) are responsible for the beneficial effects of hypercapnia following FPI.

We used staurosporine and GÖ6976 to identify the specific PKC isozyme involved in the beneficial effect of hypercapnia following FPI. Staurosporin is a nonspecific PKC inhibitor that inhibits various PKC isozymes, including PKCα, PKCβ, PKCγ, PKCδ, and PKCε [33, 53]. GÖ6976 is a relatively selective PKC inhibitor, which inhibits PKCa, PKCβ, and PKCγ. Although our study may indicate that PKCε is important for the beneficial effect of hypercapnia following FPI, a specific PKCε inhibitor εV1–2 [54] should have been used in this study, which we could not obtain in this experiment.

The model of FPI is one of the most frequently used models to mimic the clinic condition of TBI without skull fracture and has the similar histopathological changes as TBI. The severity of FPI is classified according to the values of impact force, which were controlled between 1.5 and 2 atm (classified as moderate FPI) in our study to reduce the mortality to 19.97% (data not shown).

Zhou et al. reported that in a rat model of global cerebral ischemia reperfusion injury (CIRI), PaCO_2_ of 80–100 mmHg produced a better neuroprotective effect than PaCO_2_ of 60–80 mmHg [21]. Therefore, we used PaCO_2_ of 80–100 mmHg in this experiment. Future studies will be performed to explore the beneficial effects of TH at a sub-acute time window at different duration and levels and to identify the underlying neuroprotective mechanism.

TH has been used in various brain injury animal models, and the data indicates neuroprotective effects of elevated CO_2_. Though still in the experimental period, promising laboratory studies and accumulating clinical studies suggest potential roles for clinical application of CO_2_ for patients with brain disease, preferably in avoiding the coning crisis period when first applied in the clinic. In a clinical study [55], it is reported that in patients with subarachnoid hemorrhage, hypercapnia (PCO_2_ 50–60 mmHg) was not associated with increasing of the ICP and hypercapnia could possibly be safely performed in these patients. In a clinical study from our department, continuous inhalation of CO_2_ during one-lung ventilation (OLV) improved respiratory function and mitigated the OLV-related lung and systemic inflammation in patients undergoing a lobectomy [56]. Moreover, according to the literature, patients can tolerate PaCO_2_ elevation (more than 150 mmHg according to several case reports) more than previously assumed. Such findings are in line with our results. CO_2_-related ICP proved to be reversible without permanent damage to the health of patients in these clinical reports [55, 57]. Considering that TH does not significantly alter ICP and is associated with less adverse effects in these studies, TH is now available for further study and clinical use.

## Conclusions

In summary, we found that hypercapnia (for 3 h with PaCO_2_ levels 80–100 mmHg) after TBI reduced brain edema, improved BBB function, inhibited lesion volume, and improved neurological outcome in a rat model of FPI. Hypercapnia improves neuronal function following FPI, possibly via upregulation of PKCε.

## Abbreviations

BBB: Blood–brain barrier
CBF: Cerebral blood flow
FPI: Fluid percussion injury
HR: Heart rate
ICP: Intracranial pressure
MAP: Mean arterial pressure
OLV: One-lung ventilation
TBI: Traumatic brain injury
TH: Therapeutic hypercapnia
TJ: Tight junction
